# Analysis of the Results of Tuberculosis Drug Resistance Surveillance in Yuexiu District, Guangzhou City, 2013–2022

**DOI:** 10.1002/iid3.70060

**Published:** 2024-11-27

**Authors:** Xueqiu Li, Jianxiong Liu

**Affiliations:** ^1^ Department of One Branch, Guangzhou Chest Hospital, State Key Laboratory of Respiratory Disease Guangzhou Key Laboratory of Tuberculosis Guangzhou China; ^2^ State Key Laboratory of Respiratory Disease, Guangzhou Key Laboratory of Tuberculosis, Guangzhou Chest Hospital, Institute of Tuberculosis Guangzhou Medical University Guangzhou China

## Abstract

**Introduction:**

The objectives of the study are to understand the drug‐resistant situation and trend of tuberculosis patients in Yuexiu District, Guangzhou City, from 2013 to 2022, and to provide a scientific basis for the development of rational drug‐resistant tuberculosis prevention and control strategies in Guangzhou City.

**Methods:**

All patients who were diagnosed with active tuberculosis in Guangzhou Chest Hospital from January 1, 2013 to December 31, 2022 were collected as study subjects, and a total of 5191 patients were enrolled in the study. Comprehensive data on the basic characteristics, diagnostic, and therapeutic information of the study subjects were collected. Sputum specimens were subjected to smear, isolation, and culture. Culture‐positive strains of bacteria were identified by bacterial groups. A total of 1659 strains of *Mycobacterium tuberculosis* (MTB) isolates were obtained. The drug susceptibility test was carried out using the proportionality method on the MTB isolates for nine types of antituberculosis medicines: isoniazid (INH), rifampicin (RFP), ethambutol (EMB), streptomycin (Sm), kanamycin (Km), ofloxacin (Ofx), capreomycin (Cm), propylthioisonicotinamide (Pto), and *p*‐aminosalicylic acid (PAS). A comparative analysis of the resistance patterns among the strains was conducted.

**Results:**

A total of 1659 patients with MTB were cultured, revealing 438 drug‐resistant cases. Among these, 255 were monoresistant, 121 were polyresistant, and 62 were multidrug resistant. The overall resistance rate was 26.40% (438/1659), with mono‐resistance rate at 15.37% (255/1659), polyresistance rate at 7.29% (121/1659), and multidrug resistance rate at 3.74% (62/1659). In descending order, the resistance rates of MTB isolates to any of the nine antituberculosis drugs were Sm (12.24%, 203/1659), INH (9.22%, 153/1659), EMB (7.35%, 122/1659), RFP (6.99%, 116/1659), PAS (3.25%, 54/1659), Pto (3.13%, 52/1659), Ofx (2.71%, 45/1659), Cm (2.17%, 36/1659), and Km (2.17%, 36/1659). The differences in resistance rates were statistically significant (*p* < 0.01), with Sm exhibiting the highest resistance rate and Km the lowest.

In the primary treatment group, 388 patients (25.55%) were drug resistant, while 50 patients (35.46%) in the retreatment group were drug resistant. Thirty‐nine patients (2.57%) in the primary treatment group were multidrug resistant, compared to 23 patients (16.31%) in the retreatment group. The resistance rate and multidrug resistance rate of isolates from retreatment patients were significantly higher than primary treatment patients (*p* < 0.05).

**Conclusions:**

The problem of drug‐resistant tuberculosis transmission in Guangzhou requires attention, and drug‐resistant screening should be further increased to effectively control the source of infection.

## 1 Introduction

Tuberculosis (TB) is a major infectious disease worldwide, with high morbidity and mortality rate. The global burden of multidrug‐resistant tuberculosis (MDR‐TB) continues to increase without any signs of decreasing and poses a serious threat to global TB control efforts.

In 2023, the World Health Organization (WHO) released its Global Tuberculosis Report, estimating that 10.6 million people worldwide had TB in 2022, up from 10.3 million in 2021. An estimated 410,000 people had multidrug‐ or rifampicin‐resistant TB in 2022, among whom only about two‐fifths were receiving treatment [1].

MDR‐TB has become a significant public health problem worldwide [2]. We collected demographic data and diagnostic information of active TB patients enrolled in Guangzhou Chest Hospital and the results of drug susceptibility testing (DST) of isolated strains from 2013 to 2022. We analyzed these results to understand the level of MDR‐TB and changes in Yuexiu District of Guangzhou, providing a reference for the development of TB prevention, control, and treatment policies.

## 2 Methods

### 2.1 Source of Information

All newly diagnosed patients with active TB in Guangzhou Chest Hospital from January 1, 2013 to December 31, 2022 were enrolled in the study. All enrolled participants signed the patient informed consent form, and 5191 cases were finally included. The basic conditions, diagnosis, and treatment information of the study subjects were collected, and the sputum specimens were smeared and isolated for culture. Drug susceptibility and mycobacterial group identification tests were conducted on culture‐positive strains for nine anti‐TB drugs using the proportional method, and a total of 1659 isolates of *Mycobacterium tuberculosis* (MTB) were obtained. The cohort comprised 1221 male and 438 female cases, with age distributions as follows: 72 cases aged 0–19 years, 601 cases aged 20–39 years, 576 cases aged 40–59 years, and 410 cases aged 60 years and above. Furthermore, the cohort included 1518 primary treated cases and 141 re‐treated cases.

### 2.2 Laboratory Quality Control

The laboratory staff participates in proficiency testing and inter‐room quality control activities organized by the National Tuberculosis Reference Laboratory (NTRL) for drug sensitivity testing of anti‐TB drugs each year. For each batch of samples, drug sensitivity testing the sensitive strain of MTB (H37Rv) provided by the NTRL is used as a quality control.

### 2.3 Definition of Drug‐Resistant TB

Definitions of drug‐, multidrug‐, mono‐, and polo‐resistant TB refer to the Drug‐resistant Tuberculosis Chemotherapy Guidelines (2019 short version) [3]. Definitions of primary and re‐treatment refer to Technical Guidelines for Tuberculosis Control and Prevention in China [4].

#### 2.3.1 Mono‐Resistant TB

TB patients infected with MTB have been shown to be resistant to only one first‐line anti‐TB drug by in vitro drug susceptibility testing.

#### 2.3.2 Polyresistant TB

TB patients infected with MTB confirmed by in vitro DST to be resistant to more than one first‐line anti‐TB drug (but not both isoniazid and rifampicin).

#### 2.3.3 Multidrug‐Resistant TB

TB patients infected with MTB have been shown by in vitro DST to be resistant to at least both isoniazid and rifampicin.

#### 2.3.4 Primarily Treated Patients

Patients who have not received anti‐TB treatment or have been on anti‐TB treatment for less than 1 month.

#### 2.3.5 Re‐treatment Patients

Patients on anti‐TB treatment for at least 1 month, and patients who have failed initial treatment and relapsed [4].

### 2.4 Statistical Processing

Excel 2019 software was used to organize and summarize the data, and SPSS 22.0 (IBM, Armonk, NY, USA) software was applied to analyze the data. The counting data were described by percentage (%), and the difference of drug resistance among groups was compared by chi‐square test. The trend of drug resistance over time was analyzed by the chi‐square trend test, with significance defined as *p* < 0.05.

## 3 Results

### 3.1 Drug Resistance Situation of Strains

From January 1, 2013 to December 31, 2022, MTB isolates from 5191 patients in Yuexiu District, Guangzhou City, were analyzed, identifying 438 cases of drug resistance, with a resistance rate of 26.40% (438/1659). There were 255 cases of mono‐resistance, with a mono‐resistance rate of 15.37% (255/1659); 121 cases of polyresistance, with a polyresistance rate of 7.29% (121/1659); 62 cases of multidrug resistance, with a multidrug resistance rate of 3.74% (62/1659). No statistically significant changes were observed in the drug resistance, mono‐resistance, polyresistance, or multidrug resistance rate (*p* > 0.05). Trends in resistance rates were observed, with an increase in mono‐drug and poly‐drug resistance rates and a decrease in multidrug resistance rate, although these changes did not show statistical significance (*p* > 0.05), as shown in Table 1.

**Table 1:** Drug resistance situation of *Mycobacterium tuberculosis* culture strains in Yuexiu District, Guangzhou City, 2013–2022.

		Drug‐resistance		Multidrug resistance		Mono resistance		Poly resistance	
Year	Strain number	Strain number	Drug resistance rate (%)	Strain number	Multidrug resistance rate (%)	Strain number	Mono resistance rate (%)	Strain number	Multi‐resistance rate (%)
2013	39	9	23.08	2	5.13	5	12.82	2	5.13
2014	117	28	23.93	4	3.42	17	14.53	7	5.98
2015	105	27	25.71	3	2.86	16	15.24	8	7.62
2016	182	49	26.92	6	3.30	29	15.93	14	7.69
2017	217	59	27.19	7	3.23	35	16.13	17	7.83
2018	200	57	28.50	6	3.00	33	16.50	18	9.00
2019	230	58	25.22	10	4.35	34	14.78	14	6.09
2020	197	54	27.41	8	4.06	31	15.74	15	7.61
2021	208	54	25.96	9	4.33	30	14.42	15	7.21
2022	164	43	26.22	7	4.27	25	15.24	11	6.71
Total	1659	438	26.40	62	3.74	255	15.37	121	7.29
Chi‐square value		0.016		0.209		0.134		0.388	
*p* value		0.992		0.769		0.935		0.824	

### 3.2 Distribution of Different Characteristics of Strains

In all, 1659 MTB isolates included 1221 males and 438 females. Three hundred and twenty‐four males and 115 females were found to be drug resistant, but the difference was not statistically significant (*p* > 0.05). Forty‐six males and 16 females were identified as multidrug resistant. The overall resistance rate among the 1659 MTB isolates was 26.40% (438/1659), whereas the multidrug resistance rate was 3.74% (62/1659), and the difference between the resistance rate and multidrug resistance rate of different genders was not statistically significant (*p* > 0.05). The number of drug‐resistant patients undergoing primary treatment was 388, contributing to a resistance rate of 25.55% (388/1518). In contrast, among re‐treated patients, the number was 50, yielding a resistance rate of 35.46% (50/141). Thirty‐nine patients were classified as multidrug resistant among primary treatment, equating to a rate of 2.57% (39/1518). In re‐treated patients, 23 cases exhibited multidrug resistance, constituting a higher rate of 16.31% (23/141). Both the resistance rate and multidrug resistance rate of isolates from re‐treated patients were significantly higher than those from primary‐treatment patients (*p* < 0.05), as shown in Table 2.

**Table 2:** Drug resistance situation of different characterized *Mycobacterium tuberculosis* culture strains in Yuexiu District, Guangzhou City, 2013–2022.

Characteri zation	Strain number	Drug resistance				Multidrug resistance			
		Strain number	Drug resistance rate (%)	Chi‐square value	*p* value	Strain number	Multidrug resistance rate (%)	Chi‐square value	*p* value
Gender				0.013	0.909			0.011	0.916
Male	1221	323	26.45			46	3.77		
Female	438	115	26.26			16	3.65		
Age group (years)				0.253	0.881			0.0506	0.975
0–19	72	19	26.39			3	4.17		
20–39	601	154	25.62			22	3.66		
40–59	576	156	27.08			21	3.65		
≥ 60	410	109	26.59			16	3.90		
Patient category				6.413	0.011			67.732	< 0.001
Primary treatment	1518	388	25.55			39	2.57		
Re‐treatment	141	50	35.46			23	16.31		

### 3.3 Resistance of Strains to Anti‐TB Drugs

The resistance rates of MTB isolates to the nine anti‐TB drugs, in descending order, were as follows: Sm > INH > EMB > RFP > PAS > Pto > Ofx > Cm > Km, with statistically significant differences (chi‐square value = 72.569, *p* < 0.01). Trend analysis showed statistically significant differences in the resistance rates of isolates to the three first‐line anti‐TB drugs (RFP, Sm, and EMB) across each years. The differences in the resistance rates were statistically significant, and the changes in the resistance rates of the strains to RFP, EMB, and Sm showed an increasing trend. The resistance rates to INH, Km, and PAS showed a decreasing trend, while the resistance rates to other drugs remained basically stable, as shown in Table 3.

**Table 3:** Resistance of *Mycobacterium tuberculosis* culture strains to different antituberculosis drugs in Yuexiu District, Guangzhou City, 2013–2022.

		INH resistance		RFP resistance		Sm resistant		EMB resistance		Km resistance		OFx resistance		Cm resistance		PAS resistance		Pto resistance	
Certain year	Number of strains	Number of strain	Drug resistance rate (%)	Number of strain	Drug resistance rate (%)	Number of strain	Drug resistance rate (%)	Number of strain	Drug resistance rate (%)	Number of strain	Drug resistance rate (%)	Number of strain	Drug resistance rate (%)	Number of strain	Drug resistance rate (%)	Number of strain	Drug resistance rate (%)	Number of strain	Drug resistance rate (%)
2013	39	4	10.26	2	5.13	2	5.13	2	5.13	1	2.56	1	2.56	1	2.56	2	5.13	1	2.56
2014	117	11	9.40	3	2.56	18	15.38	15	12.82	2	1.71	4	3.42	3	2.56	3	2.56	4	3.42
2015	105	9	8.57	15	14.29	4	3.81	3	2.86	2	1.90	3	2.86	2	1.90	4	3.81	3	2.86
2016	182	17	9.34	9	4.95	25	13.74	11	6.04	4	2.20	5	2.75	4	2.20	3	1.65	5	2.75
2017	217	21	9.68	17	7.83	25	11.52	18	8.29	4	1.84	6	2.76	4	1.84	7	3.23	7	3.23
2018	200	19	9.50	13	6.50	27	13.50	15	7.50	3	1.50	6	3.00	4	2.00	4	2.00	6	3.00
2019	230	21	9.13	19	8.26	31	13.48	14	6.09	5	2.17	6	2.61	5	2.17	9	3.91	8	3.48
2020	197	18	9.14	13	6.60	21	10.66	16	8.12	6	3.05	4	2.03	4	2.03	7	3.55	7	3.55
2021	208	19	9.13	14	6.73	28	13.46	15	7.21	6	2.88	6	2.88	5	2.40	8	3.85	6	2.88
2022	164	14	8.54	11	6.71	22	13.41	13	7.93	3	1.83	4	2.44	4	2.44	7	4.27	5	3.05
Total	1659	153	9.22	116	6.99	203	12.24	122	7.35	36	2.17	45	2.71	36	2.17	54	3.25	52	3.13
Chi‐square trend value		0.012		9.823		9.788		9.974		0.595		0.098		0.493		1.327		0.715	
*p* value		0.999		< 0.01		< 0.01		< 0.01		0.743		0.952		0.781		0.515		0.916	

### 3.4 Drug Resistance of Strains From Different Types of Patients

The drug resistance rate, multidrug resistance rate, and poly‐drug resistance rate of isolates from patients with re‐treatment were all higher than those from patients undergoing primary treatment, as shown in Table 4.

**Table 4:** Drug resistance of *Mycobacterium tuberculosis* culture strains in different patient types in Yuexiu District, Guangzhou City, 2013–2022.

Certain year	Primary patient culture strain									Re‐treatment patient culture strain								
	Total bacteria number of strains	Number of resistant strains	Drug resistance rate of strain (%)	Number of multidrug‐resistant strains	Multidrug resistance rate of strain (%)	Number of single drug‐resistant strains	Single drug resistance rate of strain (%)	Number of multidrug‐resistant strain	Multidrug resistance rate of strain (%)	Total bacteria number of strains	Number of resistant strains	Drug resistance rate of strain (%)	Number of multidrug‐resistant strains	Multidrug resistance rate of strain (%）	Number of single drug‐ resistant strains	Single drug resistance rate of strain (%)	Number of multidrug‐ resistant strain	Multidrug resistance rate of strain (%)
2013	33	8	24.24	1	3.03	4	12.12	1	3.03	6	2	33.33	1	16.67	1	16.67	1	16.67
2014	107	27	25.23	2	1.87	16	14.95	5	4.67	10	4	40.00	2	20.00	1	10.00	2	20.00
2015	96	25	26.04	2	2.08	14	14.58	6	6.25	9	3	33.33	1	11.11	2	22.22	2	22.22
2016	168	41	24.40	4	2.38	27	16.07	10	5.95	14	5	35.71	2	14.29	2	14.29	4	28.57
2017	199	53	26.63	4	2.01	32	16.08	12	6.03	18	6	33.33	3	16.67	3	16.67	5	27.78
2018	181	51	28.18	3	1.66	30	16.57	13	7.18	19	6	31.58	3	15.79	3	15.79	5	26.32
2019	213	51	23.94	7	3.29	31	14.55	11	5.16	17	7	41.18	3	17.65	3	17.65	3	17.65
2020	180	47	26.11	5	2.78	27	15.00	12	6.67	17	6	35.29	3	17.65	4	23.53	3	17.65
2021	193	49	25.39	6	3.36	28	14.51	12	6.22	15	5	33.33	3	20.00	2	13.33	3	20.00
2022	148	37	25.00	5	3.57	22	14.86	8	5.41	16	6	37.50	2	12.50	3	18.75	3	18.75
Chi square trend value	1518	0.0117		0.131		0.224		0.809		141	0.369		0.565		0.129		0.088	
*p* value		0.943		0.963		0.494		0.641			0.832		0.754		0.938		0.957	

## 4 Conclusion and Comment

The resistance rate of 1659 MTB isolates from patients with primary treatment was 25.55%, and 35.46% for those with re‐treatment. These rates were lower than those reported in Hebei Province, specifically 33.07% for primary treatment and 44.19% for re‐treatment [5]. The rate of multi‐drug resistance from patients with primary treatment was 2.57%, which was lower than the rate in Hebei Province (5.52%). The rate of multi‐drug resistance from patients with re‐treatment was 16.31%, which was higher than the rate in Hebei Province (6.98%).

The total resistance rate of MTB isolates was 26.40%, which was lower than the national resistance baseline survey (39.12%) and Hebei Province (34.20%), but higher than Beijing City (24.83%) [6], Shanghai Municipality (21.00%), and Fujian Province (19.04%) [7], and comparable to Gansu Province (26.26%) [8].

The multidrug resistance rate of 3.74% was lower than those reported in Beijing (5.28%), Shanghai (4.98%), Fujian (3.84%), and Gansu provinces (8.44%), as well as the national average level (4.50%) and the baseline survey on drug resistance of TB in China (8.32%) [9, 10]. It was also lower than the rate of multi‐drug resistance (4.90%) [11]. The 2007 province‐wide multi‐drug resistance baseline survey in Guangdong Province indicated that Guangzhou has made progress in monitoring MDR‐TB epidemic surveillance, preventive control, and treatment follow‐up. More patients with drug‐resistant re‐treatment may be not included in the surveillance due to seeking medical attention at higher‐level or out‐of‐province hospitals.

The rates of drug resistance and multi‐drug resistance in primary treatment patients were lower than those in re‐treatment patients, but the absolute number of drug‐resistant patients and multi‐drug resistance in primary treatment patients was much higher. Primary‐treatment patients were individuals who had never used drugs or had been using drugs for less than a month, potentially resulting in a high prevalence of primary drug resistance. A study by Meijian showed that primary drug resistance was an important reason for the emergence of drug‐resistant TB at present.

Therefore, it is particularly important to actively carry out screening for drug resistance to anti‐TB drugs, expediting the development and implementation of TB testing methods with high sensitivity and good specificity, aiming to increase the positive detection rate of pathogens. This endeavor facilitates the early identification of patients with drug‐resistant TB, thereby reinforcing the treatment of primary patients and ensuring rigorous tracking and follow‐up management of TB patients. Such measures are instrumental in bolstering treatment adherence among primary patients and increasing the cure rate.

The rates of multi‐drug resistance and drug resistance in re‐treatment patients were higher than those in primary‐treatment patients, and a history of anti‐TB treatment may serve as a risk factor for the development of drug resistance and multi‐drug resistance. It may also be related to the high medical cost, poor treatment compliance, and irregular or inappropriate medication use among re‐treatment patients. Consequently, prompt drug sensitivity testing is imperative for re‐treatment‐resistant patients, and a reasonable and effective chemotherapy regimen should be formulated based on the results of drug sensitivity testing to improve the cure rate and further mitigating the progression of drug resistance.

The rates of drug resistance and multidrug resistance were higher in men compared to women. This may be attributed to the higher prevalence of smoking and alcohol consumption among male patients. Additionally, it may be related to the greater variability in men's workplaces, which may lead to irregular treatment. Thus, it is important to enhance the frequency and rigor of tracking and follow‐up treatments for male patients.

The age group of 40–59 years old exhibited the highest drug resistance rate, which may be related to the high pressure of study, work, life, and mental stress, similar to the results from Gansu Province [8].

In addition, drug resistance posed a significant hindrance to the effective prevention and treatment of TB [12]. TB, an infectious disease that is both curable and preventable, becomes difficult to treat when resistance develops against the most effective and tolerable first‐line anti‐TB drugs [13].

The annual resistance rates of MTB strains isolated from Yuexiu District in Guangzhou City showed an overall increasing trend for RFP, EMB, and Sm, while showing an overall decreasing trend for INH, Km, and PAS. The resistance rate to Ofx (2.71%) was lower than the national level (3.49%), as well as those reported in Hunan Province (5.02%) [14] and Fujian Province (5.02%) [4]. This discrepancy may be related to the adjustment of the clinical treatment program for TB in Guangdong Province in the last 10 years, which has discontinued the use Ofx.

In summary, the problem of drug‐resistant TB in Yuexiu District, Guangzhou City, still requires great attention. The implementation of drug‐resistant screening among TB patients should be further strengthened. Timely conduct of drug sensitivity tests is essential, and reasonable and effective treatment programs should be formulated to improve the cure rates and thus mitigate the spread of drug‐resistant TB.

## Author Contributions

Conception and design: Xueqiu Li. Analyze and process data: Xueqiu Li. Drafted the manuscript: Xueqiu Li. Review and editing: Xueqiu Li and Jianxiong Liu.

## Conflicts of Interest

The authors declare no conflicts of interest.

## Supporting information

Supporting information.

## Data Availability

The authors have nothing to report.
